# Level of confidence in and endorsement of the health system among internet users in 12 low-income and middle-income countries

**DOI:** 10.1136/bmjgh-2019-002205

**Published:** 2020-08-27

**Authors:** Sanam Roder-DeWan, Anna Gage, Lisa R Hirschhorn, Nana A Y Twum-Danso, Jerker Liljestrand, Kwanele Asante-Shongwe, Talhiya Yahya, Margaret Kruk

**Affiliations:** 1 Global Health and Population, Harvard University T H Chan School of Public Health, Boston, Massachusetts, USA; 2 Health Systems, Impact Evaluation and Policy, Ifakara Health Institute, Dar es Salaam, Tanzania; 3 Medical Social Sciences, Northwestern University Feinberg School of Medicine, Chicago, Illinois, USA; 4 Maternal and Child Health, University of North Carolina Gillings School of Global Public Health, Chapel Hill, North Carolina, USA; 5 Bill and Melinda Gates Foundation, Seattle, Washington, USA; 6 African Organisation for Research and Training in Cancer, Cape Town, South Africa; 7 Quality Management Unit, Health Quality Assurance Department, Ministry of Health, Community, Development, Gender, Elderly and Children, Dodoma, Tanzania

**Keywords:** health services research, health systems

## Abstract

**Introduction:**

People’s confidence in and endorsement of the health system are key measures of system performance, yet are undermeasured in low-income and middle-income countries (LMICs). We explored the prevalence and predictors of these measures in 12 countries.

**Methods:**

We conducted an internet survey in Argentina, China, Ghana, India, Indonesia, Kenya, Lebanon, Mexico, Morocco, Nigeria, Senegal and South Africa collecting demographics, ratings of quality, and confidence in and endorsement of the health system. We used multivariable logistic regression to assess the association between confidence/endorsement and self-reported quality of recent healthcare.

**Results:**

Of 13 489 respondents, 62% reported a health visit in the past year. Applying population weights, 32% of these users were very confident that they could receive effective care if they were to ‘become very sick tomorrow’; 30% endorsed the health system, that is, agreed that it ‘works pretty well and only needs minor changes’. Reporting high quality in the last visit was associated with 4.48 and 2.69 greater odds of confidence (95% CI 3.64 to 5.52) and endorsement (95% CI 2.33 to 3.11). Having health insurance was positively associated with confidence and endorsement (adjusted odds ratio (AOR) 1.68, 95% CI 1.49 to 1.90 and AOR 1.34, 95% CI 1.22 to 1.48), while experiencing discrimination in healthcare was negatively associated (AOR 0.67, 95% CI 0.56 to 0.80 and AOR 0.63, 95% CI 0.53 to 0.76).

**Conclusion:**

Confidence and endorsement of the health system were low across 12 LMICs. This may hinder efforts to gain support for universal health coverage. Positive patient experience was strongly associated with confidence in and endorsement of the health system.

**Key questions**

**What is already known?**

- Confidence in and endorsement of (ie, agreeing that the system works well) health systems are important measures of a high-quality health system but are understudied in low-income and middle-income countries.
- Poor healthcare quality has been widely documented in these settings and may contribute to low confidence and endorsement.

**What are the new findings?**

- Our findings demonstrate low confidence in and endorsement of health systems in 12 low-income and middle-income countries.
- Reporting a recent high-quality experience and having health insurance are associated with higher health system confidence and endorsement, while negative experiences with healthcare are associated with low confidence and endorsement

**What do the new findings imply?**

- Our study countries appear to have a deficit in health system confidence and endorsement.
- This deficit may make it challenging to garner public support for healthcare reforms such as universal health coverage.

## Introduction

The Lancet Global Health Commission on High-Quality Health Systems in the Sustainable Development Goals Era (HQSS) proposed three main outcomes of a high-quality health system—better health, economic benefit and confidence in the health system.^1^ A high-quality health system provides people with a sense of security, or confidence, that they or their family members could get effective care if illness strikes. Endorsement of the health system—agreeing that the system functions well—is a summative measure that is related to, but broader than, confidence.^2 3^ Measures of endorsement are designed to challenge the respondent to think beyond their personal healthcare access and experience. Both confidence in and endorsement of the health system are valuable metrics of system performance, particularly as health systems become more oriented to the needs and values of people.^4–7^

In addition to inspiring security, confidence in the health system can prompt stronger patient engagement including appropriate health service utilisation, higher adherence to healthcare recommendations and better continuity of care, which in turn can improve health.^8–11^ Confidence can also translate into a more successful response to crises because populations that have developed trust are more likely to heed health advice and turn to the health system during emergencies—this is a feature of resilient health systems.^12^ Health system endorsement—typically operationalised as ‘the system is functioning well and only needs minor changes’—has been collected in high-income and middle-income countries and reflects respondent perceptions of health system performance at the population level.^2 3^

Despite the importance of confidence in and endorsement of health systems, these outcomes have been understudied in low-income and middle-income countries (LMIC), as have been their determinants. There is a mounting body of evidence showing that healthcare in LMICs is overall poor and highly variable across conditions, which is likely to have a deleterious effect on both confidence and endorsement. The basic foundations of healthcare are often weak; many facilities lack basic treatments and workforce shortages are widespread. Clinical encounters are often short, basic elements of clinical assessment and diagnosis are missing, and patients are not always treated with the dignity and respect that they deserve.^1 13^ These deficiencies contribute to poor health outcomes, but they are also likely to dissipate people’s confidence in and endorsement of their health systems.

In this study, we assess the level of confidence in and endorsement of the health system among internet users in 12 LMICs. Internet users (‘Internet users’ are defined here as people who are using the internet. Using the United Nations variable definition, ‘The Internet is defined as a worldwide public computer network that provides access to a number of communication services including the World Wide Web and carries email, news, entertainment and data files. Internet access may be via a computer, Internet-enabled mobile phone, digital TV, games machine, etc…’) are a growing but understudied population in LMICs. They are more educated and wealthier than the population at large and thus are not representative. However, their relatively privileged position may herald the future direction of public opinion on healthcare. Additionally, surveying this population allows for rapid feedback on health systems that, when appropriately weighted, can be used to gauge performance. We explore the role of reported care quality on confidence and endorsement. We discuss the policy and political implications of these findings in the era of universal health coverage (UHC).

## Methods

### Study design

We fielded an internet survey in 12 LMICs: Argentina, China, Ghana, India, Indonesia, Kenya, Lebanon, Mexico, Morocco, Nigeria, Senegal and South Africa. Each respondent answered a short survey on demographics, healthcare utilisation, type and quality of the respondent’s last healthcare visit, their perceptions of the health system and healthcare vignettes (online supplementary appendices 1 and 2). All respondents were geo-located through their internet protocol (IP) address. The instrument was translated by two translators from English to local languages (online supplementary appendix 3) and back-translated by native speakers. Web-users aged 18 years or older were sampled. Data were collected in August and September 2017, and the survey was closed in each country when at least 1000 surveys were completed. This sample size allowed us to achieve a margin of error of ±3% at a 95% confidence level in all study countries.

10.1136/bmjgh-2019-002205.supp1 Supplementary data

An internet survey method was chosen to rapidly reach respondents in multiple countries and to minimise social desirability bias.^14^ Internet use is becoming increasingly common in LMICs, with 40% of the world’s people having internet access in 2019 and only 24% of countries having rates below 20%.^15^ Internet users in LMICs are typically younger (18–34 vs >35 years of age), wealthier, more educated and more likely to be male than non-users.^16^ Thus, they can be seen as a more empowered advance guard in LMICs—people who likely have greater agency in health decisions and more extensive healthcare choices. We selected LMICs with internet penetrance over 20% (online supplementary appendix 4) and with broad global representation.

Internet users were sampled using Random Domain Intercept Technology (RDIT). Users who entered a non-existent or expired website name into their browser’s address bar were randomly selected to see a pop-up message inviting them to participate in the survey. RDIT has been used to study perceptions of mental illness, antivaccination sentiment and medication use.^17–19^ Entering non-existent website names is common and generates a sample that approximates the internet-using population. In an analysis of RDIT in the USA, a country with particularly detailed data on internet users, an RDIT sample was nearly identical to the national population of internet users with correlations approaching convergence for several variables: geographic distribution of users, internet service provider used, and internet use characteristics such as the number of times a site was visited.^20^ An important consideration in some countries is that RDIT is not affected by government restrictions on social media because it does not depend on those sites for distribution.

This survey employed several methods for maximising the validity and reliability of responses. It randomly varied the response order for categorical responses and moved the screen location of the question to promote attention to the question (ie, reduce repetitive clicking). The survey disallowed duplicate responses from the same IP address. It used proprietary code to detect and deselect ‘bots’—or automated entries. The method has been found to produce stable findings over time—a measure of reliability. For example, a mental health survey repeatedly conducted in India every month for over 21 months produced consistent estimates with low standard errors (SE) as did a vaccine belief survey in Ontario.^17–19^

Finally, this approach has several advantages over more commonly used internet panels and email-based solicitation of web survey participants.^21 22^ Internet panels often comprise habitual survey respondents, not casual internet users. This can lead to inattention and jadedness.^23 24^ Email-based survey invitations may be filtered out as spam, cannot be delivered anonymously and also tend to over-represent habitual survey respondents (see online supplementary appendix 5 for further discussion of methods).^21^

### Measures

Past work, including Gilson’s theoretical work on trust and confidence,^5 25^ shows that the quality of care experienced by people affects confidence and endorsement.^4^ We developed a conceptual framework (see online supplementary appendix 6) based on that work and other literature on quality measurement and reporting in LMICs.^1 26–29^ The framework was used to design our survey and study. The outcomes of interest were confidence in and endorsement of the health system. Confidence was measured with the question ‘How confident are you that if you become very sick tomorrow, you would be able to receive effective treatment from the health system?’ Confidence was defined as a response of very confident versus somewhat, not very or not at all confident. As a sensitivity analysis, we defined confidence as either very or somewhat confident. Endorsement of the health system was defined as agreeing with the statement ‘On the whole, the system works pretty well and only minor changes are necessary to make it work better’ versus the alternatives: ‘Our health care system has so much wrong with it that we need to completely rebuild it’ and ‘There are some good things in our health care system, but major changes are needed to make it work better’.

In this survey, quality of care was measured using the respondent’s rating of overall quality during their most recent outpatient care visit. High quality is defined as a rating of good, very good or excellent. The last visit was used to maximise the accuracy of recall. We also included the past experience of discrimination in the health system, defined as ever being discriminated against, hassled or made to feel inferior by a health provider or staff. Other health system variables included frequency of visits (number of outpatient care visits in the past year), having health insurance and whether the last visit was to a public or private facility.

Individual sociodemographic characteristics known to affect confidence were added to the model: age, gender, self-reported health, educational attainment and location (urban or rural). Country fixed effects controlled for unmeasured time-invariant country characteristics. Senegal was chosen as a reference category because it has the lowest gross domestic product among study countries. Finally, because there may be differences in how people across countries rate quality due to different experiences and norms, we included an anchoring vignette on quality.^29^ The vignette described a routine hypertension visit where a nurse greets the patient, introduces herself and changes the patient’s medication, but does not ask about his symptoms or check his blood pressure. We chose this vignette because it describes observable poor healthcare quality for a common condition. Respondents who consider these descriptions as adequate by rating them as good, very good or excellent quality are considered to have low expectations of quality. Vignettes describing poor-quality of care have been used to gauge respondent expectations in a variety of settings, including in LMICs.^26 30 31^

### Analysis and sample

We described the variables of interest across the sampled countries for all respondents who completed the entire survey. Only surveys that were completed through our questions of interest were included because respondents could not progress through the instrument if responses were missing. We then focused on the subsample of respondents who visited a health facility at least once in the past year and described their quality at their last visit and their confidence in and endorsement of the health system. We measured the association between the predictors described above and health system confidence and endorsement using multivariable logistic regression. This model allowed us to control for measured variables at the individual level as well as unmeasured variation between countries by including country dummy variables. Correlation at the country level was dealt with by clustering SEs. The binary outcomes make comparing different outcomes (ie, confidence vs endorsement) more readily accessible to the reader. Descriptive statistics are presented in table 1 with sampling weights. Descriptive statistics without sampling weights are included in online supplementary appendix 7. These sampling weights were created using a ranking algorithm to approximate a nationally representative sample based on the respondent’s age, gender, location (urban or rural) and education level. Age and weight targets were based on the Census Bureau’s 2017 population estimates of the country^32^; education and location weights are described in online supplementary appendix 5.

**Table 1:** Characteristics of the study sample with population weights

	Argentina		China		Ghana		India		Indonesia		Kenya		Lebanon		Mexico		Morocco		Nigeria		Senegal		South Africa		12 countries average	
	N	%	N	%	N	%	N	%	N	%	N	%	N	%	N	%	N	%	N	%	N	%	N	%		
N respondents	1153		1422		1048		1321		1179		1106		1003		1239		1124		1166		1019		1069		13 849	
**Sociodemographics**																										
Age																										
18–29	248	22	382	27	485	46	466	35	361	31	446	40	298	30	381	31	335	30	623	53	321	31	385	36	4730	34
30–49	394	34	597	42	338	32	552	42	460	39	424	38	440	44	496	40	482	43	402	35	331	33	478	45	5394	39
50+	511	44	444	31	226	22	303	23	358	30	236	21	265	26	362	29	307	27	141	12	367	36	206	19	3725	27
Female	601	52	646	45	439	42	680	51	613	52	517	47	464	46	603	49	569	51	416	36	576	57	584	55	6708	48
Rural	70	6	538	38	483	46	805	61	682	58	848	77	107	11	214	17	449	40	353	30%	599	59%	354	33	5501	40
Secondary or more education	660	57	1023	72	720	69	545	41	648	55	385	35	638	64	784	63	455	41	946	81	205	20	953	89	7963	57
Good self-rated health status	902	78	1061	75	894	85	800	61	710	60	760	69	833	83	956	77	658	59	1006	86	539	53	880	82	9998	72
**Experience with the healthcare system**																										
No visits to the health system in the past year	338	29	637	45	453	43	455	34	474	40	374	34	461	46	347	28	471	42	398	34	526	52	323	30	5256	38
N visits in the past year (mean/SD)	3.1	3.2	2.1	2.8	1.8	2.4	3.6	3.6	2.5	3.0	2.5	3.0	1.8	2.4	3.0	3.1	2.7	3.3	2.4	2.7	2.0	3.0	2.9	3.2	2.6	3.1
Last visit was to a private facility	409	50	238	30	271	46	492	57	509	72	300	41	342	63	328	37	266	41	428	56	235	48	219	29	4036	47
Has health insurance	811	70	946	67	628	60	488	37	533	45	401	36	445	44	743	60	530	47	437	37	351	34	313	29	6625	48
**Perceptions of the healthcare system**																										
Last visit was high quality	698	86	462	59	543	91	549	63	471	67	527	72	409	75	612	69	320	49	658	86	399	81	599	80	6246	73
Ever experienced discrimination	197	17	461	32	227	22	324	24	378	32	297	27	215	21	274	22	415	37	267	23	358	35	222	21	3635	26
High rating of poor quality	607	53	672	47	533	51	723	55	645	55	386	35	551	55	610	49	458	41	604	52	518	51	513	48	6819	49
Very confident could receive effective care	300	26	294	21	520	50	276	21	286	24	502	45	328	33	272	22	209	19	624	54	354	35	484	45	4449	32
Agrees system works pretty well	339	29	379	27	238	23	368	28	488	41	308	28	370	37	298	24	289	26	353	30	293	29	377	35	4100	30

The denominator is all respondents who visited a healthcare facility in the past 12 months. See online supplementary appendix 1 for the full survey instrument.

Analyses were conducted with Stata V.14.2. This study (protocol number IRB17-0907) was determined exempt by the Harvard University Human Research Protection Program. A brief consent to participate began the online survey; potential respondents who consented were able to continue with the survey and those who declined could not. We used The Strengthening the Reporting of Observational Studies in Epidemiology (STROBE) guidelines for reporting cross-sectional studies (see online supplementary appendix 8 for the checklist).

### Patients and public involvement

Patients were first involved in this study during data collection. The study is based on previous research showing poor patient experience with healthcare in LMICs.

## Results

A total of 57 786 individuals engaged with the survey by answering the first question—‘what is your age and gender?’ We included 13 849 who completed the entire survey (24% completion rate); their characteristics using population sampling weights are described in table 1. For the full sample described in table 1, 48% of respondents were female and 40% lived in a rural area. The majority (57%) had a secondary education or higher. Over a third (38%) had not visited a health facility at least once in the past year; the mean number of visits was 3.1 in the past year. The percent with health insurance varied from 29% in South Africa to 72% in Indonesia. Many respondents did not recognise poor-quality care when it was described in a vignette; nearly half of respondents gave a high rating for the anchoring vignette describing poor-quality care. Experiencing discrimination was not uncommon among the respondents; across the countries, 26% of respondents reported ever experiencing discrimination in a healthcare visit ranging from 17% in Argentina to 37% in Morocco.

**Figure 1:**
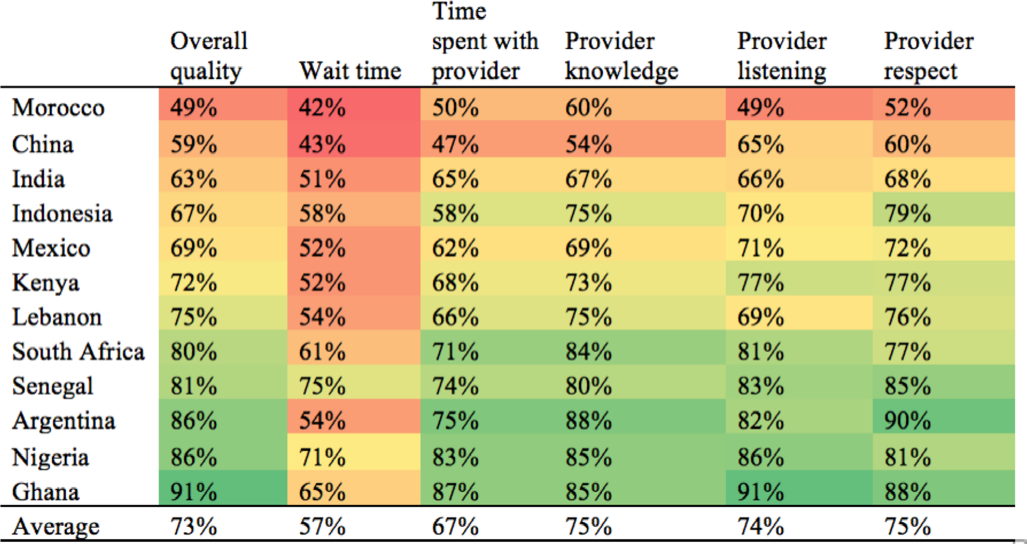
Per cent of respondents who rated quality as good, very good or excellent (n=8532). Respondents were asked to rate the quality of each element as it was experienced during their last visit to a health facility.

Figure 1 presents ratings of quality components for the most recent visit by respondents who reported facility use in the past year (8532 respondents). The demographic characteristics of this group are in online supplementary appendix 9. In most countries, a majority of respondents rated the overall quality of care as good, very good or excellent, ranging from 49% in Morocco to 91% in Ghana. Wait time and the time spent with the provider received the lowest ratings, with 57% and 67% of respondents, respectively, giving a high rating. The provider’s knowledge and level of respect they showed the respondent received the highest ratings, respectively, ranging from 54% to 85% and 52% to 90% across the countries. Because respondents were asked to rate the quality of a single visit across a variety of dimensions, quality ratings were found to be positively correlated with one another (online supplementary appendix 10).

**Figure 2:**
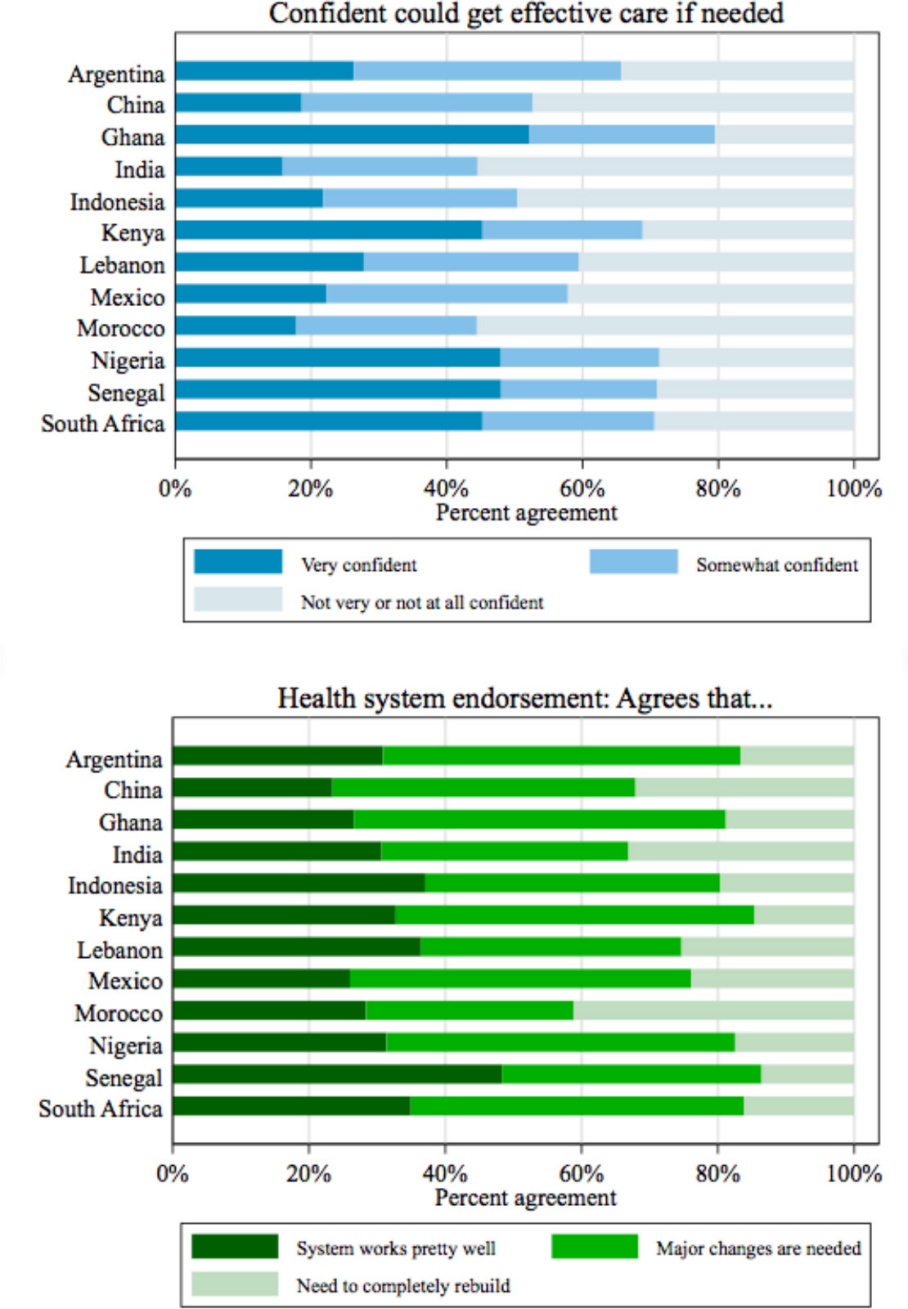
Confidence and endorsement in health system among respondents who visited a health facility in the past 12 months (n=8532).

Figure 2 shows the responses to the questions on confidence and endorsement of the health system among respondents who visited a health facility in the past year. Respondents in Ghana had the greatest confidence, with 80% of respondents indicating that they were very or somewhat confident they could receive effective treatment if they ‘become sick tomorrow’. Respondents in Morocco and India were the least confident; 45% were somewhat or very confident. The per cent of respondents that agreed with the statement ‘On the whole, the system works pretty well and only minor changes are necessary to make it work better’ ranged from 23% in China to 48% in Senegal. Across all countries, the greatest number of respondents agreed that major changes were needed. Respondents who had not visited a facility in the past year were slightly more likely to be very confident, while there was no significant difference in health system endorsement between recent visitors and non-visitors (online supplementary appendix 11).

The predictors of health system confidence and endorsement are shown in table 2. Controlling for sociodemographic characteristics, the respondent’s experience with the health system was strongly associated with their confidence and endorsement. Respondents who rated the overall quality of their last visit as good, very good or excellent had 4.48 times higher odds (95% CI 3.64 to 5.52) of stating they could get effective treatment when needed than those who rated the care as fair or poor, and 2.69 times higher odds (95% CI 2.33 to 3.11) of believing the system needed only minor changes. Health insurance was associated with 1.68 times higher odds (95% CI 1.49 to 1.90) of confidence and 1.34 times higher odds (95% CI 1.22 to 1.48) of endorsement. Experiencing discrimination was associated with lower odds of confidence and endorsement. Respondents who rated the anchoring vignette as good quality care had 2.08 and 1.43 times higher odds (95% CI 1.74 to 2.49, and 1.23 to 0.66) of being confident and endorsing the health system, respectively. Using a private facility and frequent (3+ in last year) visits to the health system were not associated with either outcome. The results were robust to lowering the threshold of confidence to somewhat or very confident; this sensitivity analysis is presented in online supplementary appendix 12.

**Table 2:** Determinants of health system confidence and endorsement among respondents who have visited a health facility in the past 12 months

	Very confident could get effective care if needed	Agrees that the health system works pretty well and only needs minor changes
	Adjusted OR (95% CI)	Adjusted OR (95% CI)
**Experience with the health system**		
Quality of last visit good, very good or excellent	**4.48 (3.64 to 5.52)**	**2.69 (2.33 to 3.11)**
Ever experienced discrimination	**0.67(0.5 to 0.80)**	**0.63 (0.53 to 0.76)**
3 or more visits to a health facility	1.03 (0.97 to 1.10)	1.03 (0.94 to 1.12)
Has health insurance	**1.68 (1.49 to 1.90)**	**1.34 (1.22 to 1.48)**
Last visit was to private facility	1.00 (0.90 to 1.12)	0.89 (0.80 to 1.00)
Vignette rating of good or better	**2.08 (1.74 to 2.49)**	**1.43 (1.23 to 1.66)**
**Sociodemographic characteristics**		
Female	0.97 (0.87 to 1.07)	0.90 (0.77 to 1.07)
Age (18–29 ref)		
30–49	0.88 (0.78 to 0.99)	**0.82 (0.73 to 0.92)**
50+	1.16 (0.9 to 1.49)	0.89 (0.79 to 1.00)
Secondary or higher education	1.09 (0.87 to 1.36)	0.83 (0.66 to 1.04)
Rural	1.33 (1.06 to 1.65)	0.99 (0.86 to 1.14)
Good self-rated health	**1.74 (1.45 to 2.10)**	**1.25 (1.10 to 1.41)**
**Country (Senegal ref)**		
Argentina	0.86 (0.81 to 0.90)	1.22 (1.18 to 1.26)
China	0.56 (0.53 to 0.59)	1.71 (1.60 to 1.82)
Ghana	**2.33 (2.20 to 2.47)**	**1.25 (1.20 to 1.30)**
India	1.07 (1.01 to 1.13)	**1.40 (1.34 to 1.46)**
Indonesia	**1.19 (1.12 to 1.25)**	**2.51 (2.36 to 2.68)**
Kenya	**1.79 (1.70 to 1.89)**	**1.60 (1.54 to 1.67)**
Lebanon	**1.24 (1.18 to 1.30)**	**2.10 (2.00 to 2.20)**
Mexico	**0.63 (0.61 to 0.65)**	**0.88 (0.85 to 0.91)**
Morocco	**0.66 (0.63 to 0.68)**	0.99 (0.94 to 1.05)
Nigeria	**3.16 (3.05 to 3.27)**	**1.38 (1.34 to 1.42)**
South Africa	**2.91 (2.76 to 3.08)**	**1.76 (1.67 to 1.85)**
N	8531	8530

Results are from multivariable logistic regressions and are in bold if p<0.001. Please see online supplementary appendix 1 for full survey instrument.

## Discussion

Confidence in the health system—defined as the belief that people can get the healthcare they need if they ‘become very sick tomorrow’— was low in this sample of 13 849 internet users in 12 countries with only one-third of respondents expressing that they were very confident. Similarly, only 30% of respondents agreed with the statement ‘On the whole, the system works pretty well and only minor changes are necessary’ with the remainder calling for major reforms or complete rebuilding of the health system. Our results are consistent with prior research. For example, a study in China reported that 28% of respondents had ‘a great deal or complete trust’ in their health system.^33^

Overall, confidence in the health system was strongly associated with the respondent’s experience with the health system. Experiencing good, very good or excellent quality care during the last visit was associated with 4.48 times greater odds of being very confident in the health system in comparison with those who reported a fair or poor-quality visit. Independent of the quality rating, we found that having ever experienced discrimination during a healthcare encounter was associated with substantially lower confidence and endorsement. This illustrates the salience of respect and equitable treatment by the health system—an emerging theme in global health discourses.^6^ Neither quantity of visits nor the type of provider (public vs private) was associated with the study outcomes. Our finding that an individual’s experience in the health system is predictive of confidence in the system is consistent with studies from high-income countries and LMICs alike.^33–35^ Macinko and colleagues^2^ found that problems in the quality of received primary care services in Latin America were associated with worse perceptions of health system performance.^2^ A study of health system confidence in the UK found that perceptions of quality at the clinical visit level had a greater influence on confidence than perceptions of the quality of the system.^34^

Although experience with the health system is most predictive of confidence in our model, individual factors are also associated with the outcome. Good self-reported health substantially increased confidence in healthcare although it had a more modest effect on endorsement. This is consistent with previous findings on trust in healthcare.^36^ The higher confidence may come from the minimisation of future risk of illness by healthy people, from having been able to obtain good care in the past, or from a positive psychological outlook or a combination.^36 37^ People who gave high-quality ratings for the poor-quality anchoring vignette were twice as likely to be confident compared with those who rated quality as fair or poor. This is reasonable because receiving effective treatment may appear more feasible to respondents with low expectations of quality or limited experience with the clinical condition in the vignette.^26^ Somewhat surprisingly people in rural areas were more confident that they could get needed care, though the effect was small (AOR 1.33, 95% CI 1.06 to 1.65). Given the challenges of internet connectivity in rural areas, those residents with internet access in rural communities may have particularly high agency and have a better knowledge of and access to the best available healthcare resources. Education (secondary or higher) was not associated with either outcome, perhaps because this was overall a well-educated group. This sample is wealthier, more educated, younger and more likely to be male than the general populations in our survey countries. It is plausible that the overall population and socioeconomically disadvantaged groups, such as those without insurance and in worse health, may have lower confidence than our study sample.

Our study has several limitations. Though the technology we used for this survey has been shown to generate samples that are representative of the internet using public,^20^ internet surveys in countries with generally low internet penetration are not representative of the full population because internet users are likely to be male, wealthy, young and more urban than the general population.^38^ This is most problematic in several low-income countries in our sample with particularly low internet penetration, such as Indonesia (20.4% internet penetration rate), Senegal (23.4%), Ghana (28.4%) and India (34.8%). The remaining countries have penetration rates upward of 45%. Internet surveys are also known to have lower response rates and potentially lower respondent attention to questions than face-to-face surveys.^39^ However, our survey had a relatively high response rate for internet surveys.^21 39^ We addressed inattention by limiting the length of the questionnaire, varying location of questions on the screen and structuring the survey for ease of response. Using only an internet survey meant that we relied on respondent reporting of health and keeping the survey short meant that we could only include the most essential questions. For example, we asked about health insurance status to capture a respondent’s protection from catastrophic health expenditures, but could not also explicitly ask about out-of-pocket expenditures. To address recall bias, we limited respondent reporting of quality to visits that occurred during the last 12 months. The resulting subpopulation may report quality differently than the full sample. However, an analysis of our outcomes of interests between those who did and those who did not have a visit in the last 12 months showed only a slightly higher likelihood of reporting confidence among those who had not had a visit and no difference in endorsement. Finally, and as is common to multicountry studies, respondents from different countries are likely to define and understand terms and questions differently. In addition to carefully structuring questions with this in mind, translating, back-translating and piloting our instrument before collecting data, we included the country in our statistical models as well as vignettes designed to measure varying expectations of healthcare quality.

The study also has several strengths. We know of only two multicountry surveys outside of high-income countries that assess these outcomes.^2 35^ The World Health Surveys and Afrobarometer surveys ask fewer questions about health systems and, in the case of the World Health Surveys, the data are now 15 years old.^4 40^ Another strength is the use of an internet survey, which allowed for a rapid assessment of sentiment and cross-national comparison. Internet surveys are particularly well suited for exploring topics prone to social desirability and acquiescence biases, as health system ratings might be since respondents can submit opinions anonymously.^41^

This research demonstrates that there is a sizeable gap in confidence and endorsement of the health system among the internet using public in these 12 LMICs. Furthermore, internet users are a relatively elite subgroup in LMICs, suggesting that these results may underestimate low health system confidence. The findings are troubling for several reasons. First, HQSS argues that confidence in health systems is one of the three primary outcomes of a high-quality health system.^1^ By this measure, each of the study countries still needs to make substantial improvements to achieve high-quality care.

Second, the perceived quality of care received during healthcare visits is the strongest predictor of confidence in our models. The implication is that improving confidence will require the hard work of improving processes of care, both technical and interpersonal, a task which will only have large impacts and be sustainable if the foundations of a health system are strengthened.^1 42^ Four universal actions for improving health system quality are recommended by HQSS: transform the workforce, ignite demand, redesign service delivery, govern for quality. Though each country will have a different balance of challenges, system strengths and system weaknesses and thus will need a unique configuration of policies and interventions addressing these universal actions,^43 44^ all countries should prioritise and plan for improved quality of care in order to address low system confidence.

Finally, populations that do not have confidence in their health systems may impede progress towards ambitious new development goals, especially UHC. Without careful planning, lack of confidence in the public health system in countries working towards UHC may divert users to poorly regulated private options, exposing them to financial hardship.^45^ Achieving quality UHC will also require the support of citizens who believe that the health system is a worthwhile use of their contributions and public resources.^46 47^

Further research on population confidence in health systems is needed and especially important as LMICs try to garner public support and funding for UHC. In addition to understanding the state of confidence in populations, scholarship on the relationships between quality healthcare processes and confidence in systems as well as the relationship between confidence and health outcomes and economic benefits of high-quality care is needed. Research that highlights the perceptions and experiences of vulnerable populations, populations that are largely excluded from this internet-based study, will be essential as countries embark on UHC.

## Conclusion

This study draws attention to a significant gap in population confidence in and endorsement of health systems in 12 LMICs. Respondents with poor-quality experiences during their last visit are more likely to lack confidence, suggesting that UHC and related efforts, such as the revitalisation of primary healthcare, should place high-quality and respectful care at their centre. Data on confidence and endorsement should be used to track the progress of health systems as they reform.
