# Supplementary material for: Level of confidence in and endorsement of the health system among internet users in 12 low-income and middle-income countries
Source: BMJ Glob Health. 2020 Aug 27;5(8):e002205. doi: 10.1136/bmjgh-2019-002205 (PMC7454186; doi:10.1136/bmjgh-2019-002205)
Supplement: Supplementary data [file bmjgh-2019-002205supp001.pdf]

Appendix: The level of confidence in and endorsement of the health system among internet users in 12 low- and middle-income countries

Appendix 1: Survey of user experience, instrument

Note: Programming instructions are in red, response options are in italics. All questions are asked to all respondents unless otherwise noted.

Harvard University is studying how to make health care better in [**Country name**]. Please help us by participating in the short voluntary, anonymous survey. Your identity is completely protected.

Question 1.
What is your age and gender?

Age/gender selection

Question 2.
In general, would you say your health is…

Excellent
Very good
Good
Fair
Poor

Question 3.
In general, how would you rate your mental health, including your mood?

Excellent
Very good
Good
Fair
Poor

Question 4.
Where do you live?

City
Town
Village

Question 5.
What is the highest level of education you have completed?

No formal schooling
Some primary school
Primary school completed
Some secondary or high school
Secondary or high school completed

Some college or university
Completed college or university

Change names in Question 7 and 8 according to country:

Country	Name
South Africa	Junior
Nigeria	Femi
Kenya	John
Senegal	Babacar
Ghana	Afram
Mexico	Juan
Argentina	Juan
India	Krishna
China	Zhang Wei
Indonesia	Panji
Lebanon	Adam
Morocco	Youssef

Question 7.

[**Anthony**] is a 45-year old man with high blood pressure who needs a regular check-up. At the health facility, the nurse
DOES: -Greet him and introduce herself -Change his medications
DOES NOT: -Ask about his symptoms -check his blood pressure

How would you rate the overall quality of care that [Anthony] received?
Poor
Fair
Good
Very Good
Excellent

Question 8.

Randomize receipt of one of the five vignettes below:

a. [**Anthony**] is a 45-year old man with high blood pressure who needs a regular check-up. At the health facility, the nurse…
DOES: -check his blood pressure -Change his medications
DOES NOT: -Greet him and introduce herself -Ask about his symptoms

b. [**Anthony**] is a 45-year old man with high blood pressure who needs a regular check-up. At the health facility, the nurse…
DOES: -Change his medications
DOES NOT: -Greet him and introduce herself -Ask about his symptoms -check his blood pressure

c. [**Anthony**] is a 45-year old man who has hurt his arm in a motorcycle accident. At the health facility, the nurse...
DOES: -Greet him and introduce herself -Wrap his arm with a bandage
DOES NOT: -Ask about his arm -Examine his arm

d. [**Anthony**] is a 45-year old man who has hurt his arm in a motorcycle accident.
At the health facility, the nurse...
DOES: -Examine his arm - Wrap his arm with a bandage
DOES NOT: -Greet him and introduce herself -Ask about his arm

e. [**Anthony**] is a 45-year old man who has hurt his arm in a motorcycle accident.
At the health facility, the nurse...
DOES: -Wrap his arm with a bandage
DOES NOT: -Greet him and introduce herself -Ask about his arm -Examine his arm

How would you rate the overall quality of care that [**Anthony**] received?

Poor
Fair
Good
Very Good
Excellent

Question 9.
In the **past year**, how many times did you go to a clinic, health center, or hospital to receive health care for yourself? (Please do not include any times you stayed overnight.)

0
1
2
3
4
5
6
7
8
9
10+

NOTE: If Q9 = 0, skip to Q19

Question 10.
What was the main reason for your last visit to a health care facility? (Excluding overnight hospital stays)

Emergency, accident, or injury
Pregnancy or delivery related
Check-up, annual exam, or preventive care
Chronic existing problem
New health problem or question

Question 11.
Who managed the last facility that you visited for care (excluding overnight hospital stays)?

Government
Private

Show prompt below for Questions 12-16

Thinking about your last visit to a health care facility (not including overnight stays), how would you rate the following:

Randomize order of Questions 12-16

Question 12.
The length of time you waited before you were seen.

Poor
Fair
Good
Very Good
Excellent

Question 13.
Whether the provider listened carefully to you.

Poor
Fair
Good
Very Good
Excellent

Question 14.
Provider's medical knowledge and skills.

Poor
Fair
Good
Very good
Excellent

Question 15.
The level of respect the provider showed you.

Poor
Fair
Good
Very good
Excellent

Question 16.
The amount of time the provider spent with you in the visit.

Poor
Fair
Good
Very good
Excellent

Question 17.
Overall, taking everything into account, how would you rate the quality of care you received?

Poor
Fair
Good
Very good
Excellent

Question 18.
Overall, thinking about your entire visit, how satisfied were you with the care you received?

Very dissatisfied
Somewhat dissatisfied
Somewhat satisfied
Very satisfied

Question 19.
In the last year, was there a time when you had a medical problem and needed medical attention, but you did not visit a health facility?

Yes
No
No medical problems

NOTE: If Q19=No or No medical problems, skip to Q21

Question 20.
What is the main reason you did not visit a health facility?
What there another reason you did not visit a health facility?
What there another reason you did not visit a health facility?

Randomize list of response options below. Allow respondents to choose option at a time, re-randomize, and ask second question.

None of the above
Too far/no transport
Costs are too high
Drugs/supplies not available
Health care providers lack knowledge/skills
Wait times are too long
Health care staff are disrespectful

Question 21.
Have you ever been discriminated against, hassled, or made to feel inferior by a health provider/staff for any of these reasons?

- Poverty
- Religion
- Ethnicity, Language
- Immigration, Migrant status
- Because of your gender
- Type of Illness
- Education
- Romantic or Sexual attraction to someone of the same sex
- Any other reason

Yes
No

Question 22.
You mentioned that you were discriminated against by health providers/staff. What were the reasons? Choose as many as apply.

Poverty
Religion
Ethnicity, Language
Immigration, Migrant Status
Education
Romantic or sexual attraction to someone of the same sex
Type of illness
Because I am a man/woman
Other

Question 23.
How confident are you that if you become very sick tomorrow, you would be able to receive effective treatment from the health system?

Not at all confident
Not very confident
Somewhat confident
Very confident

Question 24.
With which statement do you agree most?

Our health care system has so much wrong with it that we need ***to completely rebuild it****.*
There are some good things in our health care system, but ***major changes*** *are needed to make it work better.*
On the whole, the system works pretty well and only ***minor changes*** *are necessary to make it work better.*

Question 25.
Do you have health insurance? (Any service, either from the government or a private company, that pays for some or all of your health care costs)

Yes
No

End survey with note below:

Thank you for participating in this survey. We appreciate your time.

Appendix 2: Survey instrument screen shot

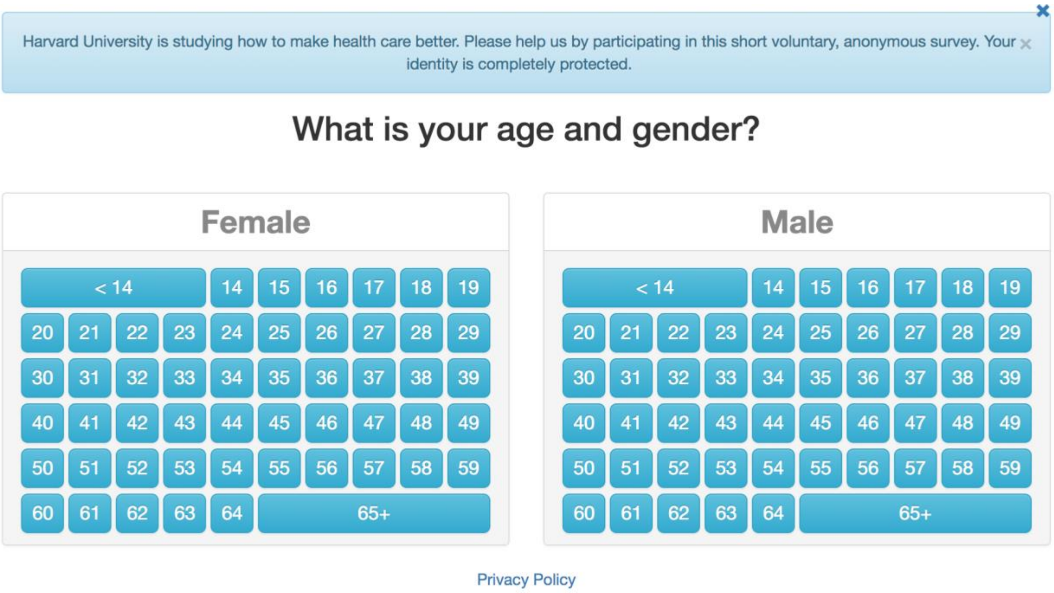

Appendix 3: Survey Languages[a]

Country	Survey Language
Senegal	Senegalese French
Ghana	English
Kenya	English
India	English[b]
Nigeria	English
Morocco	Moroccan Arabic
Indonesia	Bahasa
S. Africa	English
Lebanon	Lebanese Arabic
China	Chinese
Mexico	Mexican Spanish
Argentina	Argentine Spanish

a – Languages were chosen based on most commonly spoken language in country
b - Previous experience with internet surveys in India by the RIWI Corp showed that, when given a choice, Indian internet respondents overwhelmingly selected English

Appendix 4: Internet penetration rates in survey countries

Country	Internet penetration (2016)
Senegal	23.4%
Ghana	28.4%
Kenya	45.0%
India	34.8%
Nigeria	46.1%
Morocco	57.6%
Indonesia	20.4%
South Africa	52.0%
Lebanon	75.9%
China	52.2%

Mexico	45.1%
Argentina	69.2%

www.internetlivestats.com

Appendix 5: Expanded methods

Reproducibility
In a study of global mental health stigma[1], researchers tested the reproducibility of responses produced by RDIT[tm] sampling methods. A short survey was fielded every month for 21 consecutive months in India. Responses were very similar across the survey months; 31.1 +/- .18% s.e. replied yes to the question, "is there someone you interact with every day who suffers from mental illness?" An average of 10.1 +/- .11% s.e. said that "people who suffer from mental illness are more violent." The group also compared global responses excluding India between June and July of 2014 and June and July of 2015 and found no statistically significant differences for any of their questions.

Habitual respondents
A study of RDIT[tm] in 2014[2] asked 50,313 respondents the following question: "prior to this survey when was the last time you answered survey questions?" 72% of respondents had not taken a survey in the last month and 47% of respondents had never taken a survey before.

Representativeness
An independent research group analyzed the cookies produced by the RDIT[tm] sample over the course of one week in 2013[3] and found highly statistically significant correlations with characteristics of the U.S. internet using population. The group matched a total of 115,000 unique cookies. Almost complete convergence (correlations between .915 and .997) was found between the two samples when looking at the internet service providers, geographic distribution, number of cookie hits and age of the cookies.

Anchor vignettes
Anchoring vignettes can be included in surveys that compare self-reported health across countries or groups of people that are likely to have differing norms and expectations. The anchoring vignette allows researchers to control for these factors, identify comparable cut-off points between ordinal response categories, and make interpersonal comparisons despite social difference in response characteristics.

Sampling weight construction
The age and gender weight targets were created based on the Census Bureau's 2017 Population Estimates of the country's population. The urban and rural targets were created based on the Central Intelligence Agency database. The education targets for most countries were created based on educational attainment data from the UNESCO Institute for Statistics. Education targets for Nigeria were created based on data from the Demographic and Health Survey conducted by the National Population Commission of Nigeria and education targets for Morocco were based on educational enrolment data from the High Commission for the Plan of Morocco.

Appendix 6: Conceptual framework

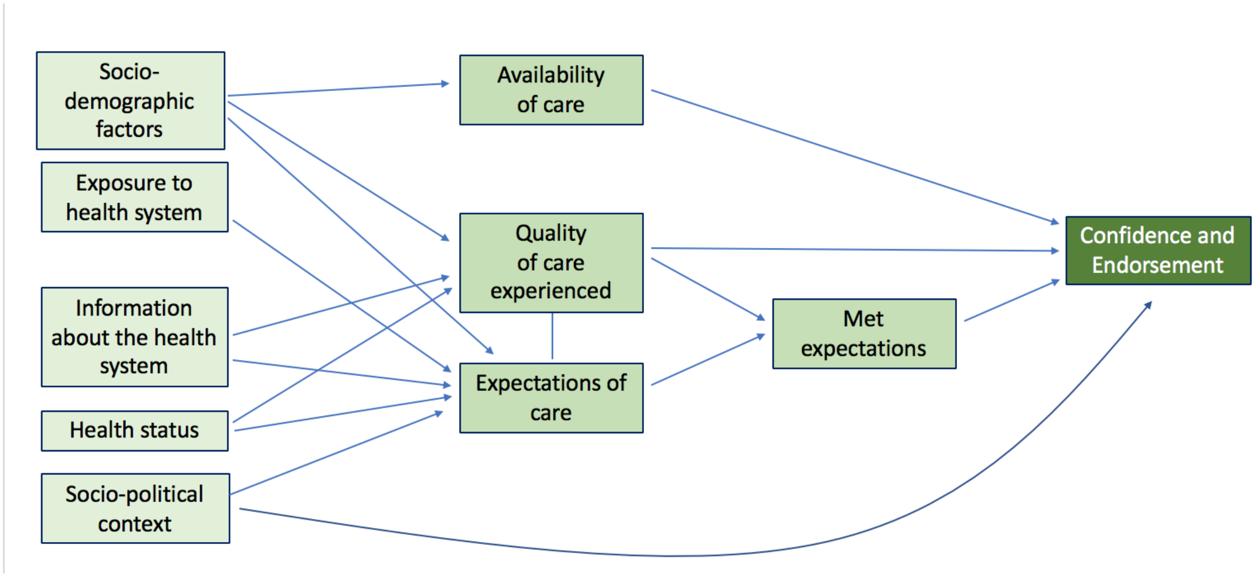
Socio-demographic factors
Exposure to health system
Information about the health system
Health status
Socio-political context
Availability of care
Quality of care experienced
Expectations of care
Met expectations
Confidence and Endorsement

Appendix 7: Sample characteristics without population weights

	Argentina		China		Ghana		India		Indonesia		Kenya		Lebanon		Mexico		Morocco		Nigeria		Senegal		South Africa		Total	
	N	%	N	%	N	%	N	%	N	%	N	%	N	%	N	%	N	%	N	%	N	%	N	%	N	%
N respondents	1,153		1,422		1,048		1,321		1,179		1,106		1,003		1,239		1,124		1,166		1,019		1,069		13,849	
Socio-demographics																										
Age: 18-29	471	41%	858	60%	715	68%	879	67%	758	64%	693	63%	559	56%	656	53%	667	59%	777	67%	598	59%	627	59%	8258	60%
30-49	438	38%	456	32%	279	27%	360	27%	356	30%	342	31%	338	34%	398	32%	340	30%	336	29%	319	31%	370	35%	4332	31%
50+	244	21%	108	8%	54	5%	82	6%	65	6%	71	6%	106	11%	185	15%	117	10%	53	5%	102	10%	72	7%	1259	9%
Female	419	36%	276	19%	251	24%	294	22%	288	24%	351	32%	275	27%	432	35%	326	29%	283	24%	288	28%	476	45%	3959	29%
Rural	157	14%	220	15%	70	7%	199	15%	245	21%	204	18%	164	16%	161	13%	118	10%	47	4%	71	7%	260	24%	1916	14%
Secondary or more education	958	83%	1232	87%	997	95%	1211	92%	1103	94%	102<br>1	92%	782	78%	1077	87%	954	85%	1132	97%	896	88%	102<br>0	95%	12383	89%
Good self-rated health status	952	83%	1079	76%	940	90%	1097	83%	839	71%	984	89%	877	87%	1031	83%	844	75%	1084	93%	822	81%	918	86%	11467	83%
Experience with health care system																										
Did not visit health system in past year	328	28%	643	45%	358	34%	466	35%	541	46%	353	32%	504	50%	377	30%	568	51%	412	35%	430	42%	337	32%	5317	38%
N visits in past year (mean/sd)	2.9	3.0	2.0	2.8	2.0	2.5	2.4	2.9	1.7	2.5	2.2	2.6	1.7	2.6	2.6	2.9	1.7	2.5	2.4	2.7	1.6	2.1	2.7	3.0	2.2	2.7
Last visit was to private facility*	469	57%	240	31%	328	48%	638	75%	345	54%	449	60%	334	67%	315	37%	267	48%	427	57%	265	45%	224	31%	4301	50%
Has health insurance	801	69%	1011	71%	748	71%	597	45%	679	58%	584	53%	507	51%	782	63%	543	48%	350	30%	417	41%	313	29%	7332	53%
Perceptions of health care system																										
Quality of last visit good, very good, or excellent*	690	84%	527	68%	632	92%	694	81%	444	70%	628	83%	402	81%	649	75%	295	53%	673	89%	457	78%	609	83%	6700	79%
Ever experienced discriminati on	201	17%	386	27%	231	22%	356	27%	288	24%	215	19%	216	22%	256	21%	364	32%	218	19%	167	16%	208	19%	3106	22%
Vignette rating of good or better	512	44%	662	47%	471	45%	737	56%	586	50%	391	35%	532	53%	563	45%	389	35%	574	49%	437	43%	505	47%	6359	46%
Very confident could receive effective care	295	26%	330	23%	520	50%	450	34%	338	29%	432	39%	362	36%	255	21%	171	15%	606	52%	270	26%	523	49%	4552	33%
Agrees system works pretty well	353	31%	539	38%	342	33%	451	34%	520	44%	380	34%	407	41%	284	23%	216	19%	377	32%	248	24%	390	36%	4507	33%

*Denominator is respondents who visited a health care facility in the past 12 months. See appendix 1 for full survey instrument.

Appendix 8: STROBE Statement—Checklist of items that should be included in reports of cross-sectional studies

	Item No	Recommendation	Section, paragraph
**Title and abstract**	1	(*a*) Indicate the study's design with a commonly used term in the title or the abstract	Title
		(*b*) Provide in the abstract an informative and balanced summary of what was done and what was found	Abstract
**Introduction**			
Background/rationale	2	Explain the scientific background and rationale for the investigation being reported	Introduction Para 1-4
Objectives	3	State specific objectives, including any prespecified hypotheses	Introduction Para 3
**Methods**			
Study design	4	Present key elements of study design early in the paper	Methods Para 1-10
Setting	5	Describe the setting, locations, and relevant dates, including periods of recruitment, exposure, follow-up, and data collection	Methods Para 1
Participants	6	(*a*) Give the eligibility criteria, and the sources and methods of selection of participants	Methods Para 1-2
Variables	7	Clearly define all outcomes, exposures, predictors, potential confounders, and effect modifiers. Give diagnostic criteria, if applicable	Methods Para 6-8
Data sources/ measurement	8*	For each variable of interest, give sources of data and details of methods of assessment (measurement). Describe comparability of assessment methods if there is more than one group	Methods Para 6-8
Bias	9	Describe any efforts to address potential sources of bias	Methods Para 8
Study size	10	Explain how the study size was arrived at	Methods Para 1
Quantitative variables	11	Explain how quantitative variables were handled in the analyses. If applicable, describe which groupings were chosen and why	Methods Para 6-8
Statistical methods	12	(*a*) Describe all statistical methods, including those used to control for confounding	Methods Para 8-10
		(*b*) Describe any methods used to examine subgroups and interactions	Methods Para 9
		(*c*) Explain how missing data were addressed	Results N/A
		(*d*) If applicable, describe analytical methods taking account of sampling strategy	Methods Para 9
		(*e*) Describe any sensitivity analyses	Results Para 4

**Results**			
Participants	13*	(a) Report numbers of individuals at each stage of study—eg numbers potentially eligible, examined for eligibility, confirmed eligible, included in the study, completing follow-up, and analysed	Results Para 1
		(b) Give reasons for non-participation at each stage	Results Para 1
		(c) Consider use of a flow diagram	
Descriptive data	14*	(a) Give characteristics of study participants (eg demographic, clinical, social) and information on exposures and potential confounders	Results Para 1
		(b) Indicate number of participants with missing data for each variable of interest	n/a
Outcome data	15*	Report numbers of outcome events or summary measures	Results Para 3
Main results	16	(*a*) Give unadjusted estimates and, if applicable, confounder-adjusted estimates and their precision (eg, 95% confidence interval). Make clear which confounders were adjusted for and why they were included	Results Para 3-4
		(*b*) Report category boundaries when continuous variables were categorized	Results Para 4
		(*c*) If relevant, consider translating estimates of relative risk into absolute risk for a meaningful time period	n/a
Other analyses	17	Report other analyses done—eg analyses of subgroups and interactions, and sensitivity analyses	Results Para 3-4
**Discussion**			
Key results	18	Summarise key results with reference to study objectives	Discussion Para 1
Limitations	19	Discuss limitations of the study, taking into account sources of potential bias or imprecision. Discuss both direction and magnitude of any potential bias	Discussion Para 4
Interpretation	20	Give a cautious overall interpretation of results considering objectives, limitations, multiplicity of analyses, results from similar studies, and other relevant evidence	Discussion Para 6-8
Generalisability	21	Discuss the generalisability (external validity) of the study results	Discussion Para 4
**Other information**			
Funding	22	Give the source of funding and the role of the funders for the present study and, if applicable, for the original study on which the present article is based	End matter

*Source: http://www.equator-network.org/reporting-guidelines/strobe/

Appendix 9: Weighted analytic sample characteristics, excluding respondents who had not visited a health facility in the last year

	Argentina		China		Ghana		India		Indonesia		Kenya		Lebanon		Mexico		Morocco		Nigeria		Senegal		South Africa		Total	
	N	%	N	%	N	%	N	%	N	%	N	%	N	%	N	%	N	%	N	%	N	%	N	%	N	%
N respondents	825		779		690		855		638		753		499		862		556		754		589		732		8532	
Socio-demographics																										
Age: 18-29	163	26%	197	29%	303	52%	316	39%	186	28%	327	46%	137	29%	278	36%	196	34%	407	54%	175	37%	279	40%	2964	38%
30-49	271	43%	300	44%	230	40%	399	49%	255	39%	340	48%	232	48%	347	45%	287	50%	286	38%	236	50%	322	47%	3506	45%
50+	193	31%	193	28%	47	8%	95	12%	213	33%	44	6%	110	23%	149	19%	88	15%	65	9%	63	13%	88	13%	1348	17%
Female	403	49%	419	53%	252	42%	475	55%	393	56%	372	51%	280	52%	453	51%	405	62%	280	36%	285	58%	405	54%	4421	51%
Rural	50	6%	315	40%	197	33%	499	58%	448	64%	553	76%	61	11%	149	17%	294	45%	216	28%	241	49%	276	37%	3299	38%
Secondary or more education	486	60%	509	65%	454	76%	330	38%	337	48%	274	37%	337	62%	550	62%	231	35%	637	83%	132	27%	667	89%	4944	58%
Good self-rated health status	632	77%	582	74%	518	87%	512	59%	450	64%	487	66%	426	79%	664	74%	343	53%	635	83%	365	74%	594	80%	6206	72%
Experience with healthcare system																										
N visits in past year (mean/sd)	4.4	3.0	3.9	2.7	3.2	2.4	5.5	3.1	4.1	2.8	3.8	3.0	3.3	2.5	4.1	2.9	4.6	3.1	3.7	2.6	4.1	3.2	4.1	3.0	4.1	3.0
Last visit was to private facility	409	50%	238	30%	271	46%	492	57%	509	72%	300	41%	342	63%	328	37%	266	41%	428	56%	235	48%	219	29%	4036	47%
Has health insurance	594	73%	544	69%	402	68%	308	36%	327	46%	280	38%	237	44%	546	61%	324	50%	318	41%	280	57%	235	31%	4395	51%
Perceptions of healthcare system																										
Quality of last visit good, very good or excellent	698	86%	462	59%	543	91%	549	63%	471	67%	527	72%	409	75%	612	69%	320	49%	658	86%	399	81%	599	80%	6246	73%
Ever experienced discrimination	165	20%	334	43%	154	26%	242	28%	292	41%	206	28%	152	28%	229	26%	229	35%	213	28%	126	25%	176	24%	2517	29%
Vignette rating of good or better	447	55%	384	49%	316	53%	469	54%	408	58%	270	37%	275	51%	454	51%	281	43%	404	53%	359	73%	362	49%	4428	52%
Very confident could receive effective care	214	26%	146	19%	311	52%	137	16%	154	22%	332	45%	150	28%	199	22%	117	18%	368	48%	237	48%	338	45%	2703	31%
Agrees system works pretty well	251	31%	184	23%	158	27%	266	31%	261	37%	240	33%	198	36%	232	26%	185	28%	241	31%	238	48%	261	35%	2715	32%

Appendix 10: **Quality rating correlation matrix**

	Overall quality	Wait time	Provider knowledge	Provider listening	Time spent with provider	Provider respect
Overall quality	1					
Wait time	0.63	1				
Provider knowledge	0.66	0.61	1			
Provider listening	0.67	0.57	0.66	1		
Time spent with provider	0.66	0.64	0.68	0.68	1	
Provider respect	0.65	0.58	0.70	0.68	0.67	1

Appendix 11: **Comparison of confidence and endorsement by use of the health system in the past year**

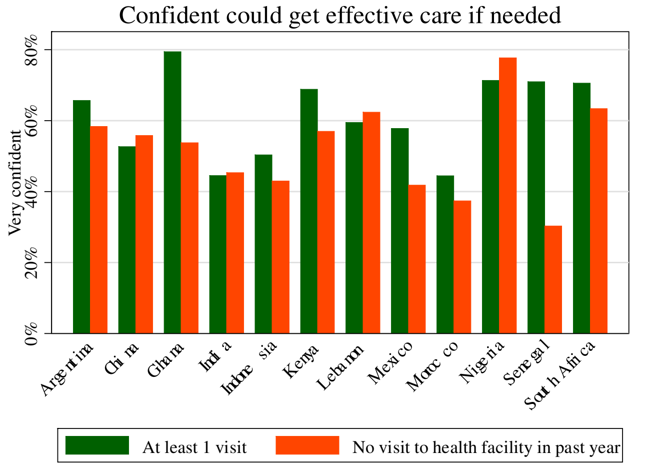

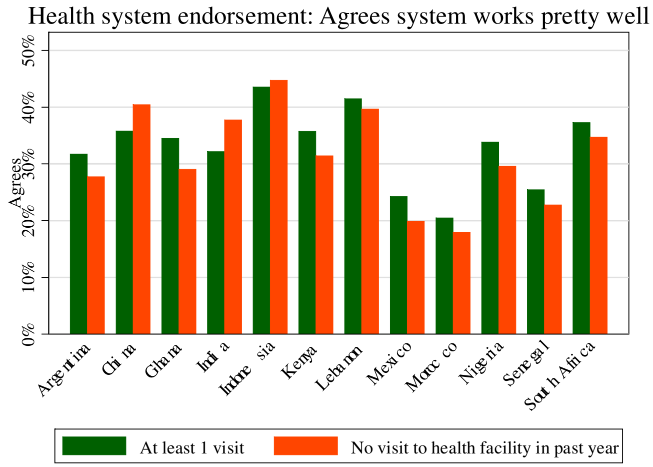

Appendix 12: Sensitivity analysis using response options of "Somewhat confident or "Very confident"

	Somewhat or very confident	
	Odds Ratio	95% CI
Experience with health system		
Quality of last visit good, very good, or excellent	4.10	[3.57,4.71]
Ever experienced discrimination	0.50	[0.43,0.59]
3 or more visits to health facility	0.98	[0.89,1.08]
Has health insurance	2.26	[1.99,2.58]
Last visit was to private facility	0.99	[0.87,1.11]
Vignette rating of good or better	1.56	[1.29,1.88]
Socio-demographic characteristics		
Female	0.91	[0.82,1.01]
Age (18-29 ref)		
30-49	0.92	[0.85,1.00]
50+	1.16	[0.88,1.53]
Secondary or higher education	1.26	[1.06,1.49]
Rural	0.88	[0.76,1.02]
Good self-rated health	1.44	[1.27,1.64]
Country (Senegal ref)		
Ghana	1.56	[1.50,1.63]
Kenya	1.55	[1.49,1.61]
India	1.05	[1.01,1.09]
Nigeria	1.74	[1.65,1.83]
Morocco	0.55	[0.53,0.58]
Indonesia	1.15	[1.10,1.20]
South Africa	1.98	[1.90,2.05]
Lebanon	0.98	[0.95,1.01]
China	1.14	[1.07,1.21]
Mexico	0.66	[0.64,0.69]
Argentina	0.83	[0.79,0.87]
N	8531	

Works Cited

1. Seeman N, Tang S, Brown AD, Ing A. World survey of mental illness stigma. *Journal of Affective Disorders* 2016; **190**: 115-21.
2. Murphy L., Pospichal L., Liebenson D., Meerkamper E. GRIT Consumer Participation in Research Report: A Global Study of Habitual vs. Non-Habitual Survey Takers. New York, NY: Greenbook and RIWI, 2014.
3. ComScore. Representativeness of the RIWI Sampling Methodology in the United States, 2014.
